# Development of a loop‐mediated isothermal amplification‐lateral flow dipstick (LAMP‐LFD) assay and on‐site rapid detection evaluation for *Rodentibacter heylii* and *Rodentibacter pneumotropicus*

**DOI:** 10.1002/ame2.70214

**Published:** 2026-05-28

**Authors:** Huiqiong Yan, Sisi Chen, Yuhan Gan, Jing Xing, Yufang Feng, Xin Wen, Jie Fang, Jiangtao Du, Shasang Zhou, Honggang Guo, Xiaoyin Jin, Zhiyuan Wang, Junhao Tao, Lingqun Lu, Qingming Kong, Huazhong Ying, Wei Han, Fangwei Dai

**Affiliations:** ^1^ Center of Laboratory Animal Hangzhou Medical College Hangzhou Zhejiang China; ^2^ Zhejiang Provincial Key Laboratory of Laboratory Animals and Safety Research Hangzhou Medical College Hangzhou Zhejiang China; ^3^ Department of Pathology, Cancer Prevention and Treatment Institute of Chengdu Chengdu Fifth People's Hospital (The Second Clinical Medical College, Affiliated Fifth People's Hospital of Chengdu University of Traditional Chinese Medicine) Chengdu Sichuan China; ^4^ Savaid Stomatology School Hangzhou Medical College Hangzhou Zhejiang China; ^5^ National Rodent Laboratory Animal Resources Center, Institute for Laboratory Animal Resources National Institutes for Food and Drug Control (NIFDC) Beijing China; ^6^ Key Laboratory of Biomarkers and In Vitro Diagnosis Translation of Zhejiang Province, School of Laboratory Medicine and Bioengineering Hangzhou Medical College Hangzhou Zhejiang China; ^7^ Engineering Research Center of Novel Vaccine of Zhejiang Province Hangzhou Medical College Hangzhou Zhejiang China; ^8^ Zhejiang Key Laboratory of High‐level Biosafety and Biomedical Transformation Hangzhou Medical College Hangzhou China

**Keywords:** LAMP‐LFD, novel detection method, *Rodentibacter heylii*, *Rodentibacter pneumotropicus*

## Abstract

**Background:**

*Rodentibacter pneumotropicus* and *Rodentibacter heylii* are important exclusion pathogens in specific pathogen‐free (SPF) laboratory animals. However, routine detection methods such as traditional bacterial culture remain labor‐intensive and time‐consuming. The loop‐mediated isothermal amplification‐lateral flow dipstick (LAMP–LFD) assay provides a self‐contained format for nucleic acid testing that combines speed, sensitivity, and specificity while minimizing aerosol contamination, making it well‐suited for point‐of‐need applications.

**Objectives:**

This study aimed to develop a streamlined, on‐site detection platform capable of rapidly identifying both pathogens with high analytical accuracy, operational simplicity, and suitability for routine laboratory surveillance.

**Methods:**

A LAMP–LFD system was established for the detection of the two pathogens using primers designed against the conserved 16S–23S rRNA ITS region. The assay's specificity, sensitivity, repeatability, and limit of detection (LoD) were evaluated.

**Results:**

The assay achieved detection limits of 1 × 10^−4^ ng/μL for *R. pneumotropicus* and 1 × 10^−5^ ng/μL for *R. heylii*, demonstrating significantly higher sensitivity than PCR. No cross‐reactivity was observed with 15 non‐target bacteria. Among 28 infected samples, the method detected 19 positives, compared with 21 by multiplex PCR and 23 by qPCR. In 842 laboratory animal samples, culture‐based, qPCR, and LAMP–LFD detected 2, 41, and 37 positive samples, respectively. The LoDs were 10^2^ and 10^1^ CFU/mL in fecal samples and 10^1^ and 10^0^ CFU/mL in throat swab samples for *R. pneumotropicus* and *R. heylii*, respectively.

**Conclusions:**

This method provides an approach for monitoring the health status of laboratory mice by enabling the detection of these two target pathogens.

## Graphical abstract

This study established a closed‐tube LAMP‐LFD assay for detecting *Rodentibacter heylii* and *Rodentibacter pneumotropicus*. The method exhibited high sensitivity (detection limits of 10^−5^ ng/μL for *R. heylii* and 10^−4^ ng/μL for *R. pneumotropicus*), high specificity, reliable performance with clinical samples, and effective validation in experimental infection models. This aerosol‐proof system requires 50 min and no specialized equipment, ideal for basic laboratories.

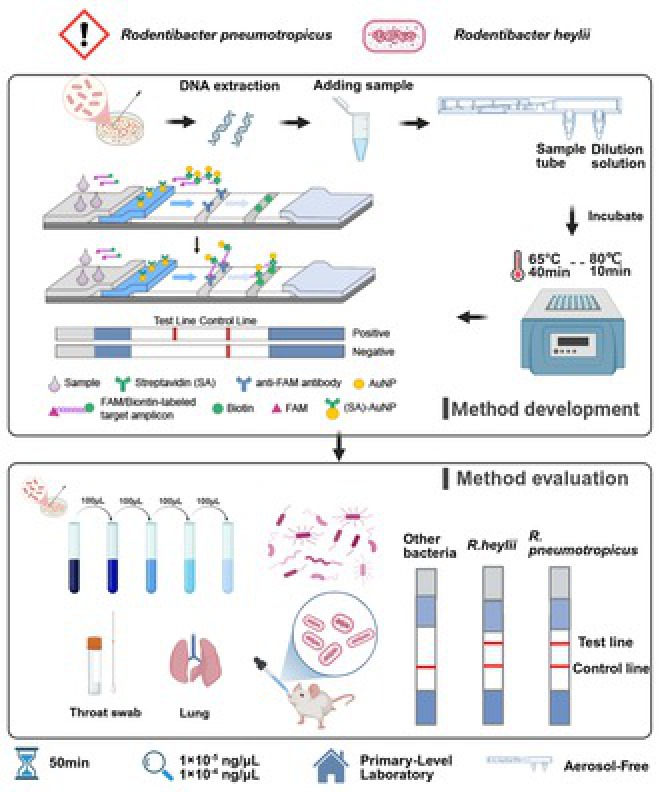

## 1 INTRODUCTION

As gram‐negative organisms, *Rodentibacter heylii* and *Rodentibacter pneumotropicus* are facultative colonizers of animal mucosae. They are ecologically established in the respiratory, reproductive, and gastrointestinal tracts across diverse species, including wildlife, livestock, and laboratory animals. While often commensal, they are opportunistic pathogens capable of producing suppurative lesions in various organs.^1^ Historically categorized as the Jawetz and Heyl biotypes, these bacteria were reclassified in 2017 into the genus *Rodentibacter*, which now includes several species such as *R. pneumotropicus* and *R. heylii*.^2^

Maintaining the microbiological quality of laboratory animal populations is essential for preserving the integrity of biomedical research. This is accomplished through comprehensive health‐monitoring programs in standard facilities, often involving sentinel animals and the testing of environmental or direct samples. The conditional pathogens *R. heylii* and *R. pneumotropicus* are critical targets within these programs and are included among the mandatory tests for specific pathogen‐free (SPF) animals.^3^ A major concern in murine infection models is the presence of confounding variables, an issue that is further amplified in studies employing immunodeficient mice, where the host response is inherently altered.^4^

Despite its status as the gold standard, traditional bacterial culture has several inherent limitations, including labor‐intensive procedures, limited specificity, suboptimal detection rates, and reduced accuracy during the early stages of infection.^5^ Although skilled personnel can differentiate target species from co‐infecting bacteria, the method's operational complexity, subjectivity, and dependence on trained expertise impede rapid decision‐making and restrict its use in large‐scale epidemiological surveys or primary‐level testing facilities.

Alternative molecular and serological methods—including PCR, multiplex PCR, qPCR, MALDI‐TOF MS, and ELISA—have been developed for pathogen detection.^1^, ^6^, ^7^, ^8^, ^9^ While PCR and qPCR offer high sensitivity and specificity, their reliance on costly equipment and trained personnel limits their suitability for routine clinical or community‐level implementations. Similarly, existing LAMP assays often rely on gel electrophoresis, turbidimetry, or fluorescent dyes, all of which are prone to non‐specific amplification interference and typically require additional instruments for result interpretation, thereby increasing the risk of false‐positive outcomes.

Loop‐mediated isothermal amplification (LAMP) is a novel isothermal technique that achieves multiplex target amplification through an auto‐cycling strand‐displacement reaction using four to six specific primers. Isothermal amplification at 60–65°C is driven by the intrinsic strand‐displacement activity of *Bst* DNA polymerase.^10^ This method is known for its simplicity and has been widely applied in the detection of bacteria, viruses, and parasites.^11^, ^12^, ^13^, ^14^, ^15^, ^16^ However, issues such as aerosol contamination and challenges in visual interpretation can limit its utility.

To address these limitations, we integrated LAMP into a closed‐tube lateral flow dipstick (LFD) format. The LFD detects biotin‐ and FAM‐labeled LAMP amplicons through a straightforward colorimetric change on the test strip, enabling visual, in situ readout while minimizing the impact of non‐specific amplification. This LAMP–LFD workflow has been successfully applied across multiple pathogen detection systems.^16^, ^17^, ^18^, ^19^, ^20^, ^21^, ^22^, ^23^ Given these advantages, LAMP is well‐positioned to become a widely adopted technique in this field. The use of a closed device further prevents aerosol contamination during sample handling and amplification, thereby improving reliability and accelerating result acquisition.^19^ This approach is particularly suitable for primary screening in grassroots experimental animal facilities.

For designing species‐specific primers, a commonly targeted locus is the well‐established genetic marker within the 16S–23S rRNA region, known as the internal transcribed spacer (ITS), owing to its characteristic combination of intra‐species conservation and inter‐species sequence divergence.^6^, ^24^, ^25^, ^26^ Consequently, we focused on constructing primers directed against the ITS sequences of the two pathogens. Our strategy centered on an integrated closed‐tube platform that couples LAMP and LFD techniques, aiming to provide a simple, reliable, and contamination‐resistant method for routine monitoring of these critical pathogens.

## 2 MATERIALS AND METHODS

### 2.1 Bacterial isolation and genomic DNA extraction

We first revived the two pathogens by culturing them on Columbia blood agar (CBA) plates and incubating the plates in a 37°C humidified incubator (GHP‐9270N, Shanghai) for 24 h. Single colonies were then transferred into Brain Heart Infusion (BHI) medium, which was prepared by supplementing BHI with 10% (v/v) heat‐inactivated, sterile‐filtered horse serum, following a previously described procedure.^1^ All additional bacterial species listed in Table 1 were cultured on Luria–Bertani, selenite enrichment, Columbia blood, or MacConkey agar media, under standardized time and temperature conditions. Genomic DNA was extracted strictly according to the protocol provided with the TIANGEN kit (DP302‐02). DNA concentration and purity were determined using a spectrophotometer (Thermo Fisher Scientific, USA) and assessed by the A260/A280 ratio. All sample concentrations were normalized to 10 ng/μL and stored at −20°C for subsequent analysis.

**TABLE 1:** Strains used in this study.

Bacteria	Source
*R. heylii* (R.H)	ATCC12555
*R. pneumotropicus* (R.P)	ATCC35149
*Shigella flexneri*	CGMCC1.1868
*Salmonella typhimurium*	CGMCC1.1194
*Salmonella enterica* subsp. *enterica serovar* *Pullorum*	ATCC13036
*Salmonella enterica*	ATCC15611
*Popoff serovar choleraesuis*	ATCC10708
*Salmonella enterica*	ATCC13314
*Corynebacterium kutscheri*	CMCC65013
*Klebsiella oxytoca*	Isolate (Zhejiang Vital River Laboratory Animal Technology Co., Ltd., Cavia porcellus)
*Pasteurella multocida*	Isolate (National Institutes for Food and Drug Control)
*Pseudomonas aeruginosa*	Isolate (Hangzhou Medical College)
*Listeria monocytogenes*	Isolate (Zhejiang University)
*Muribacter muris*	Isolate (Zhejiang University)
*Edwardsiella tarda*	Isolate (National Institutes for Food and Drug Control)
*Salmonella enterica* subsp. *enterica serovar* *Paratyphi A*	Isolate (Hangzhou Red Cross Hospital)
*Aeromonas hydrophila*	Isolate (National Institutes for Food and Drug Control)

### 2.2 Design of primers and probes

**FIGURE 1:**
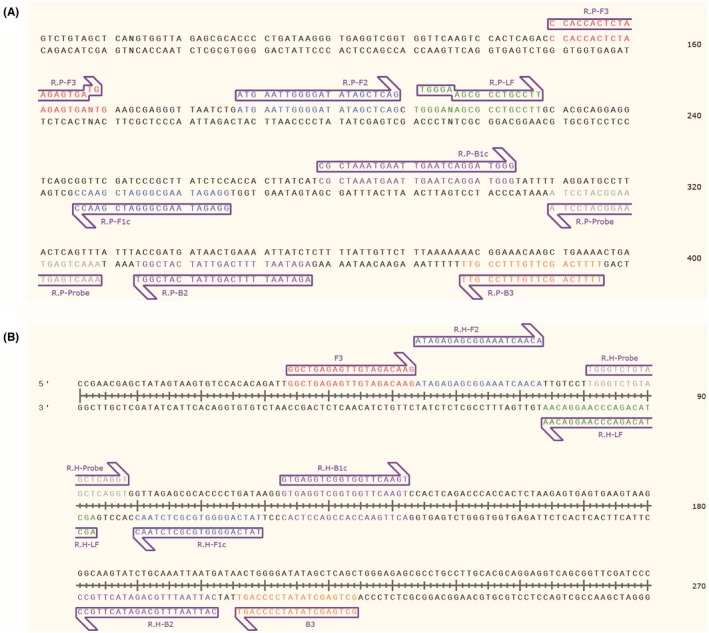
Nucleotide sequences of *Rodentibacter pneumotropicus* (A) and *Rodentibacter heylii* (B) showing the set of primers used. The internal primer FIP/BIP is composed of F1c and B1c and the complementary sequences of F2 and B2. The forward inner primer (FIP) was labeled with biotin at the 5′ end, and amplification proceeded in the 5′ → 3′ direction.

The ITS sequences targeting *R. heylii* (JX010710.1) and *R. pneumotropicus* (JX010706.1) were retrieved and downloaded from NCBI, and primers were designed based on their alignment results. Primer Explorer V5 was employed to generate LAMP primer sets from these regions (Figure 1A,B). All primers were synthesized by Tsingke Biotech (Hangzhou, China). The sequences of all primers and probes used in this study, along with their corresponding names, are detailed in Table 2.

**TABLE 2:** Primers and probes used for the detection of *R. pneumotropicus* and *R. heylii*.

	Primers	Primer sequences (5′ → 3′)
LAMP	R.H‐F3	GGCTGAGAGTTGTAGACAAG
	R.H‐B3	GCTGAGCTATATCCCCAGT
	R.H‐FIP	TATCAGGGGTGCGCTCTAAC‐ATAGAGAGCGGAAATCAACA
	R.H‐BIP	GTGAGGTCGGTGGTTCAAGT‐CATTAATTTGCAGATACTTGCC
	Bio‐R.H‐FIP	Bio‐TATCAGGGGTGCGCTCTAAC‐ATAGAGAGCGGAAATCAACA
	R.H‐LF	AACAGGAACCCAGACATCGA
	R.H‐probe	FAM‐TGGGTCTGTAGCTCAGGT
	R.P‐F3	CCACCACTCTAAGAGTGATG
	R.P‐B3	TTTTCAGCTTGTTTCCGTT
	R.P‐FIP	GGAGATAAGCGGGATCGAACC‐ATGAATTGGGGATATAGCTCAG
	R.P‐BIP	CGCTAAATGAATTGAATCAGGATGG‐GAGATAATTTTCAGTTATCATCGGT
	Bio‐R.P‐FIP	Bio‐GGAGATAAGCGGGATCGAACC‐ATGAATTGGGGATATAGCTCAG
	R.P‐LF	ACCCTTCGCGGACGGAA
	R.P‐probe	FAM‐TAGGATGCCTTACTCAGTTT
qPCR	PP‐F	GATGTGGGTGTCTCTGTAG
	PP‐R	CCATCCGRCTCGTTTCATC
	PP‐Probe	FAM‐ACCAACAGGTAGCGTAACAGTGGGTTATGGTCAG‐BHQ1
Multiplex PCR	P‐F	ATGGGAGTGGGTTGTACCA
	R.P‐R	GGCATCCTAAAATACCCATCC
	R.H‐R	TTGCAGATACTTGCCCTTAC

Abbreviations: B3, reverse outer primer; BIP, reverse inner primer; F3, forward outer primer; FIP, forward inner primer; LF, loop primer; PCR, polymerase chain reaction; R.H, *Rodentibacter heylii*; R.P, *Rodentibacter pneumotropicus*.

### 2.3 Assembly of the 25 μL LAMP reaction mixture

The mixture contained 2.5 μL of 10 × reaction buffer (NEB, #B9004S), 1.6 mmol/L dNTPs (Sangon Biotech, #B500055‐0500), 1 μL of 8 U *Bst* 2.0 DNA polymerase (NEB, #M0537S), and 6 mmol/L MgSO_4_ (NEB, #B1003S). Final working concentrations of the primers and probe were 5 μmol/L for F3 and B3, 40 μmol/L for biotinylated FIP and BIP, 20 μmol/L for LF, and 10 μmol/L for the FAM‐labeled probe. The final reaction volume was adjusted to 25 μL by adding the template DNA and nuclease‐free water.

The LFD component was incorporated into a sealed, self‐contained detection device, allowing direct visualization of results through an observation window. The test required a 40‐min incubation at 65°C. First, the diluted buffer was added to the tube, and the sample was loaded into the chamber. The incubation was then initiated. After the reaction, heat inactivation was performed at 80°C for 10 min. The contents of the two compartments were then gently mixed by turning the device, enabling the solution to migrate along the angled tube wall. The final readout appeared within approximately 10 min.

### 2.4 LAMP‐LFD assay

**FIGURE 2:**
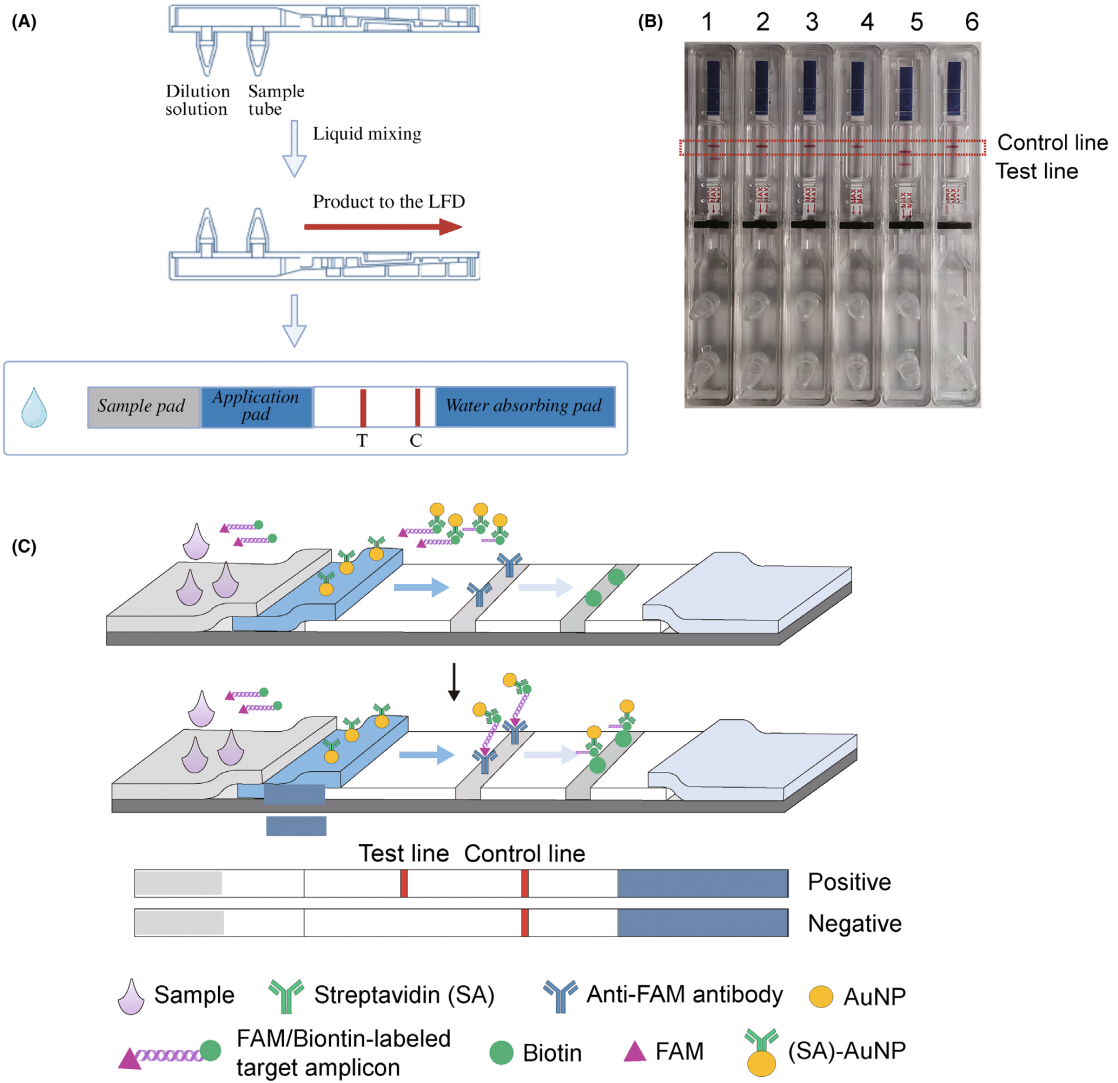
The closed device (A) A schematic representation of the closed device. (B) A graphical display of the loop‐mediated isothermal amplification‐lateral flow dipstick (LFD) results. (C) The principles of LFD.

A biotin‐modified forward inner primer (FIP) and a carboxyfluorescein (FAM)‐labeled probe were employed in the assay. The test line of the strip was immobilized with an anti‐FAM monoclonal antibody, and gold–streptavidin (SA) complexes served as the capture reagents. During amplification, the FAM‐tagged probe hybridized with the target product, forming a labeled complex. As this complex migrated along the strip, it first bound to the colloidal gold–streptavidin conjugate, which facilitated its capture at the test line by the specific anti‐FAM antibody, generating a visible signal. The dual‐labeled product was thus captured and visualized at the test line following interaction with the gold–streptavidin conjugate. Color development exclusively at the control line indicated a negative result, while coloration at both the test and control lines signified a positive result. Signals appearing solely at the test line were considered invalid. A schematic representation of the closed device is shown in (Figure 2A), the LFD readout is presented in (Figure 2B), and the principle of LFD is illustrated in (Figure 2C).

### 2.5 Performance assessment of LAMP and LAMP‐LFD: Specificity and sensitivity

The evaluation of detection specificity was performed following standard experimental procedures, with assessments conducted separately for the LAMP and LAMP‐LFD methods, as described below. Initially, the bacterial strains listed in Table 1 were preliminarily screened. This was followed by a more extensive specificity evaluation involving 62 strains, collected from multiple geographical locations, including Beijing and Shanghai. The collection comprised two pathogens and several closely related *Bartonella* species isolated from rodents (Table 3).

**TABLE 3:** Specificity of the LAMP‐LFD assay for detecting the closely related species *R. pneumotropicus* and *R. heylii*.

Bacterial strain	Source	No. of strains	LAMP–LFD result
*Rodentibacter heylii*	Isolated strain (Guangzhou, Mouse)	1	P
*Rodentibacter pneumotropicus*	Isolated strain (Guangzhou, Rat)	5	P
*Rodentibacter heylii*	Isolated strain (Shanghai, hamster)	2	P
*Rodentibacter pneumotropicus*	Isolated strain (Nanchang, Mouse)	2	P
*Rodentibacter heylii*	Isolated strain (Beijing, Mouse)	18	P
*Rodentibacter heylii*	Isolated strain (Beijing, Rat)	5	P
*Rodentibacter pneumotropicus*	Isolated strain (Beijing, Mouse)	15	P
*Rodentibacter pneumotropicus*	Isolated strain (Beijing, Gerbil)	1	P
*Rodentibacter heylii*	Isolated strain (Beijing, Guinea pig)	1	P
*Rodentibacter pneumotropicus*	Isolated strain (Beijing, Rat)	2	P
*Rodentibacter heylii*	ATCC12555	1	P
*Rodentibacter ratti*	Isolated strain (Shanghai, Rat)	1	N
*Pasteurellaceae bacterium*	Isolated strain (Shanghai, Rat)	2	N

Abbreviations: N, negative; P, positive.

For sensitivity testing, the genomic DNA of two pathogens was serially diluted 10‐fold (from 1 ng/μL to ~10^−8^ ng/μL). Conventional PCR was performed in a parallel experiment using the Premix Taq™ system (TaKaRa, RR902A). The PCR reaction mixture contained 10 μmol/L each of the F3 and B3 primers, template DNA, and nuclease‐free water. Amplification was conducted under the following conditions: an initial denaturation at 95°C for 5 min, followed by 35 cycles of 95°C, 52°C, and 72°C (each for 30 s), and a final extension at 72°C for 10 min. The amplified products were analyzed using 1.5% agarose gel electrophoresis. All amplification procedures were conducted on a C1000 Touch Thermal Cycler (Bio‐Rad, USA), and amplicons were detected using the Bio‐Rad gel imaging system.

Positive controls consisted of *R. heylii* and *R. pneumotropicus* DNA, while diethyl pyrocarbonate (DEPC) water was used as the negative control. The experimental results confirmed that both LAMP and LAMP‐LFD assays showed excellent performance, with their high specificity and sensitivity being successfully validated.

### 2.6 Establishment of the infection model

Mice were purchased from a facility holding the Laboratory Animal Production License no. SCXK (Zhejiang) 2019–0002. The animals were maintained under a 12‐h light/dark cycle at 24 ± 2°C and 40%–60% relative humidity, with food and water provided ad libitum. A total of 30 8‐week‐old SPF mice (C57BL/6 and BALB/c strains) were randomly assigned to control and infected groups. The experiments were conducted in a biosafety level 2 (ABSL‐2) laboratory (SYS330112540005). All mice underwent a three‐day acclimation period in the facility environment before the initiation of the model. Bacterial strains were initially cultured on CBA at 37°C in a 10% carbon dioxide (CO_2_) atmosphere. For secondary culture, samples were transferred into BHI medium and incubated under the same temperature and CO_2_ conditions.^1^ The animals were anesthetized using tribromoethanol. Experimental infection was performed via intranasal inoculation with 3 × 10^7^ colony‐forming units (CFU) of *R. heylii* in 20 μL of PBS (10 μL per nostril). The control group received PBS only. The animals' conditions were monitored twice daily. Euthanasia was performed when the following conditions were observed: signs of hemorrhage, a loss of 20% of initial body weight, cyanosis, or acute respiratory distress. Mice were sacrificed under pentobarbital anesthesia. Animals were anesthetized for the collection of whole blood, serum, alveolar lavage fluid, trachea, lung, liver, and kidney tissues. The study was conducted in full compliance with animal welfare regulations. For cytokine analysis, bronchoalveolar lavage fluid (BALF) was collected and subsequently analyzed for TNF‐α, IL‐1β, and IL‐1α levels using ELISA kits (JHN80229, JHN80300, JHN80536). For histopathological assessment, lung and other organ tissues were grossly examined, fixed in 4% phosphate‐buffered formalin, and subsequently processed for paraffin embedding, sectioning, and conventional hematoxylin and eosin (H&E) staining. At the conclusion of the experiment, trachea and lung tissues were isolated and stored at −80°C for long‐term preservation. DNA was then extracted from these pulmonary and tracheal tissues using a commercial kit (TIANGEN DP302, Beijing, China).

### 2.7 Clinical application of LAMP–LFD testing

To evaluate the applicability of the LAMP–LFD assay for routine monitoring, 842 lung tissue samples were collected from 23 institutions, including universities, research institutes, companies, and hospitals, representing broad geographic coverage. The samples included 18 laboratory animal strains, such as ICR, C57BL/6, BALB/c, KM mice, SD rats, and sentinel mice (Table S1). All assays were performed by the same personnel in a single laboratory using standardized protocols.

### 2.8 Comparative analysis of infected samples

We developed a multiplex PCR assay using the ready‐to‐use Ex Taq 2.0 master mix (dye version) for multiplex PCR. The reaction mixture was prepared as follows: in a 25 μL reaction system, each primer was added to a final concentration of 0.2 μmol/L, 20 ng of template DNA was included, and the volume was adjusted with sterile water. The amplification program consisted of an initial denaturation at 95°C for 5 min, followed by 30 cycles of 94°C for 1 min, 58°C for 30 s, and 72°C for 30 s, with a final extension at 72°C for 4 min.^25^ After amplification, the products were separated by electrophoresis on a 1.5% agarose gel to verify amplification success, yielding distinct bands of 326 bp for *R. heylii* and 451 bp for *R. pneumotropicus*. For quantitative PCR, the primer concentrations were 0.9 μmol/L for PP‐F and PP‐R, and the PP probe was included at 0.2 μmol/L in 1 × TaqMan probe premix, along with template DNA and deionized water. The reaction program comprised the following: 95°C for 20 s; followed by 40 cycles of 95°C for 1 s and 60°C for 20 s.^27^

### 2.9 Preparation and analysis of simulated samples

To validate the method's LoD, simulated samples were prepared by artificially contaminating mouse fecal and throat swab samples with pre‐prepared bacterial suspensions of two pathogens at graded concentrations, ranging from 10^−1^ to 10^4^ CFU/mL, with three replicates for each concentration. Detection was performed using the established LAMP‐LFD reaction system to determine the LoD. Genomic DNA was extracted from the bacterial samples following the manufacturers' protocols (Solarbio, YZ‐D4015‐01; TIANGEN, DP322‐02) and stored at −20°C until analysis.

### 2.10 Data analysis

To verify the reliability of the newly developed method for two pathogens, we used SPSS 24.0 (IBM) for statistical analysis. The *p*‐values and kappa coefficients were calculated to determine the level of agreement among the results of these diagnostic tests.

### 2.11 Ethics statement

This study was conducted in accordance with ethical guidelines for animal experimentation. All procedures were approved by the Laboratory Animal Welfare Committee of the Zhejiang Provincial Laboratory Animal Center (Approval No. ZJCLA‐IACUC‐20020119) and were strictly performed in compliance with the provisions of the NIH Guide for the Care and Use of Laboratory Animals (1996 revision).

## 3 RESULTS

### 3.1 Optimization of the LAMP system

The optimal dNTP concentration for the LAMP systems of both pathogens was determined to be 1.6 mmol/L, based on the earliest appearance of the amplification curve (Figure S1A,C). Similarly, a final Mg^2+^ concentration of 6 mmol/L produced the earliest detectable amplification in the LAMP reaction system (Figure S1B,D). After optimization, the optimal reaction temperatures for *R. pneumotropicus* and *R. heylii* at which positive amplification curves appeared earliest were established as 63°C and 64°C, respectively (Figure S2). Consequently, these concentrations and temperatures were adopted as the optimal conditions for their respective systems.

### 3.2 Specificity and sensitivity of LAMP

**FIGURE 3:**
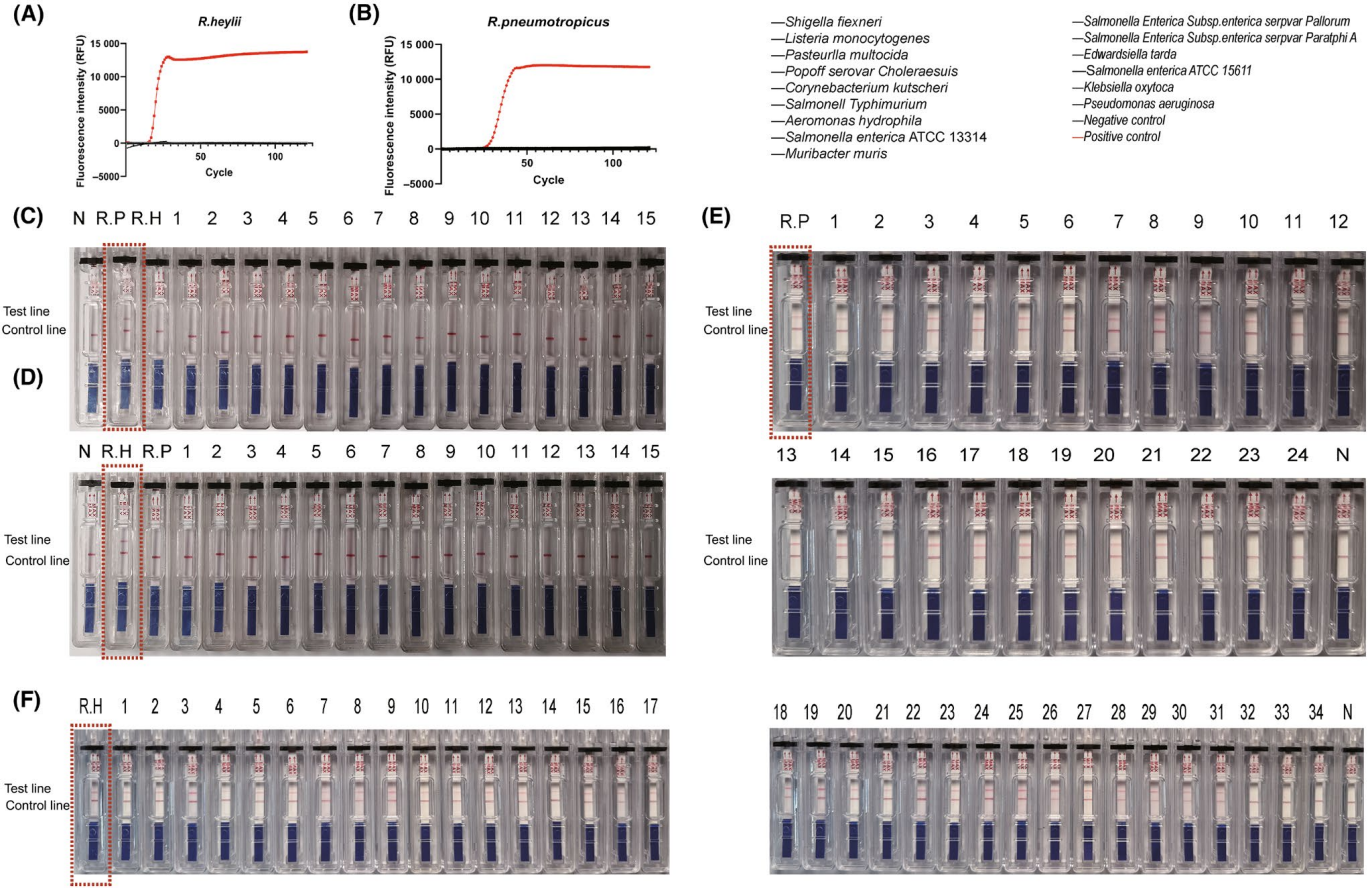
Specificity testing for *Rodentibacter heylii* and *Rodentibacter pneumotropicus* R.P, *Rodentibacter pneumotropicus*; R.H, *Rodentibacter heylii*; N, negative control. Samples 1–15 consisted of genomic DNA from *Shigella flexneri*, *Salmonella Typhimurium*, *Salmonella enterica Popoff serovar Choleraesuis*, *Salmonella enterica*, *Corynebacterium kutscheri*, *Salmonella enterica* subsp. *enterica serovar Pullorum*, *Klebsiella oxytoca*, *Pasteurella multocida*, *Pseudomonas aeruginosa*, *Listeria monocytogenes*, *Aeromonas hydrophila*, *Edwardsiella tarda*, *Salmonella enterica* subsp. *enterica serovar Paratyphi* A, and *Muribacter muris*. (A) *R. heylii* loop‐mediated isothermal amplification (LAMP) specificity. (B) *R. pneumotropicus* LAMP specificity. (C) *R. pneumotropicus* LAMP‐lateral flow dipstick (LFD) specificity. (D) *R. heylii* LAMP‐LFD specificity. (E) Analytical specificity of the LAMP–LFD assay for the detection of *Rodentibacter pneumotropicus*. Samples 1–21 correspond to *R. pneumotropicus*, sample 22 corresponds to *Rodentibacter ratti*, and samples 23–24 correspond to *Pasteurellaceae* bacterium. (F) Analytical specificity of the LAMP–LFD assay for the detection of *Rodentibacter heylii*. Samples 1–31 correspond to *R. heylii*, sample 32 corresponds to *Rodentibacter ratti*, and samples 33–34 correspond to *Pasteurellaceae* bacterium.

**FIGURE 4:**
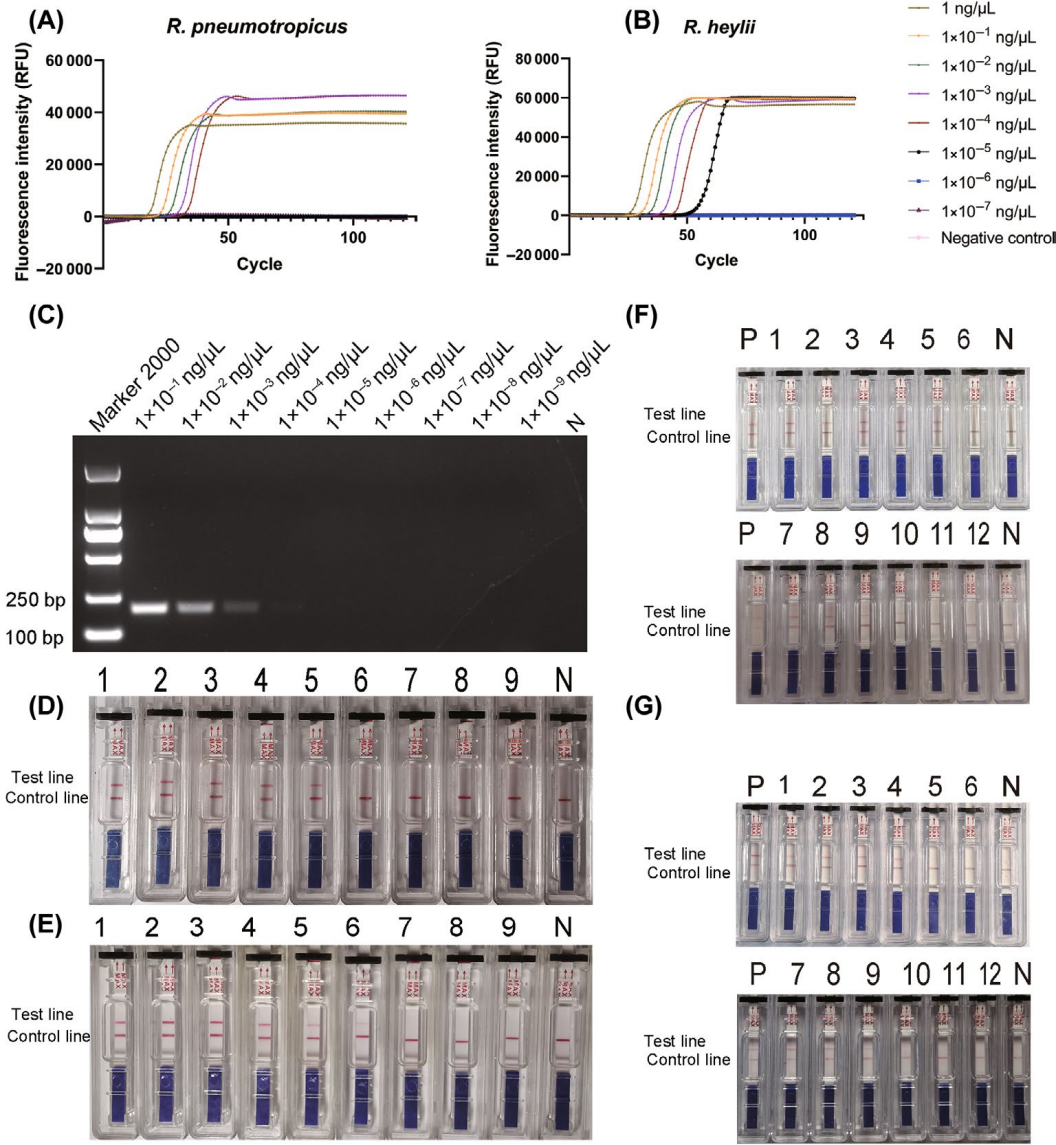
*Rodentibacter heylii* and *Rodentibacter pneumotropicus* sensitivity tests. N: negative control; 1–9 correspond to 1 ng/μL, 1 × 10^−1^ ng/μL, 1 × 10^−2^ ng/μL, 1 × 10^−3^ ng/μL, 1 × 10^−4^ ng/μL, 1 × 10^−5^ ng/μL, 1 × 10^−6^ ng/μL, 1 × 10^−7^ ng/μL, and 1 × 10^−8^ ng/μL. (A) Loop‐mediated isothermal amplification (LAMP) sensitivity for *R. pneumotropicus*. (B) LAMP sensitivity for *R. heylii*. (C) Conventional polymerase chain reaction (PCR) sensitivity assay showing single amplicons of the expected size. Unlike LAMP reactions, which typically generate ladder‐like concatemer patterns, PCR amplification produces a single specific band. (D) LAMP–lateral flow dipstick (LFD) sensitivity for *R. pneumotropicus*. (E) LAMP–LFD sensitivity for *R. heylii*. (F) Samples 1–6: fecal samples containing *R. heylii* at concentrations of 10^4^, 10^3^, 10^2^, 10^1^, 10^0^, and 10^−1^ CFU/mL; samples 7–12: throat samples at the same concentrations. (G) Samples 1–6: fecal samples containing *R. pneumotropicus* at concentrations of 10^4^, 10^3^, 10^2^, 10^1^, 10^0^, and 10^−1^ CFU/mL; samples 7–12: throat samples at the same concentrations.

The results showed that, with the exception of *R. heylii* and *R. pneumotropicus*, none of the bacteria listed in Table 1 displayed amplification curves (Figure 3A,B). DNA was diluted for sensitivity testing, ranging from 1 ng/μL to 1 × 10^−7^ ng/μL. Amplification curves were observed at a concentration of 1 × 10^−5^ ng/μL (Figure 4A,B), establishing the sensitivity of the LAMP method at 1 × 10^−5^ ng/μL. In comparison, the LoD of the PCR method was determined to be 1 × 10^−4^ ng/μL (Figure 4C).

### 3.3 Sensitivity and specificity of LAMP‐LFD

The test was conducted using an integrated containment device to prevent aerosol generation (Figure 2). The results for *R. heylii*, *R. pneumotropicus*, and related strains collected from Beijing, Shanghai, Nanchang, Guangzhou, Hangzhou, and other locations were further validated using the LAMP‐LFD method. Assay specificity was evaluated using *R. heylii*, *R. pneumotropicus*, *Rodentibacter ratti*, and other rodent‐associated members of the *Pasteurellaceae* family (Table 3; Figure 3C,D). All closely related non‐target species, including *R. ratti* (samples 22 and 32) and *Pasteurellaceae* bacterium (samples 23–24 and 33–34), yielded negative results. Only *R. heylii* and *R. pneumotropicus* produced positive amplification signals, confirming the analytical specificity of the assay (Figure 3E,F). DNA concentrations of the two pathogens were serially diluted over a range from 1 ng/μL to 1 × 10^−7^ ng/μL. The results demonstrated that the detection sensitivity of the LAMP‐LFD method for the two targets was 1 × 10^−4^ ng/μL and 1 × 10^−5^ ng/μL, respectively (Figure 4D,E). The fastest results could be obtained within 15 min by optimizing the reaction interval (data not shown).

### 3.4 Detection of simulated clinical samples

We further evaluated the detection performance of the LAMP‐LFD test kit using simulated fecal and throat swab samples artificially spiked with serial dilutions of the two pathogens. Following DNA extraction, the assays were performed as described above. The LoD for *R. heylii* and *R. pneumotropicus* were 10^1^ and 10^2^ CFU/mL in throat swab samples, and 10^0^ and 10^1^ CFU/mL in fecal samples, respectively (Figure 4F,G).

### 3.5 Results of the infection model and samples

**FIGURE 5:**
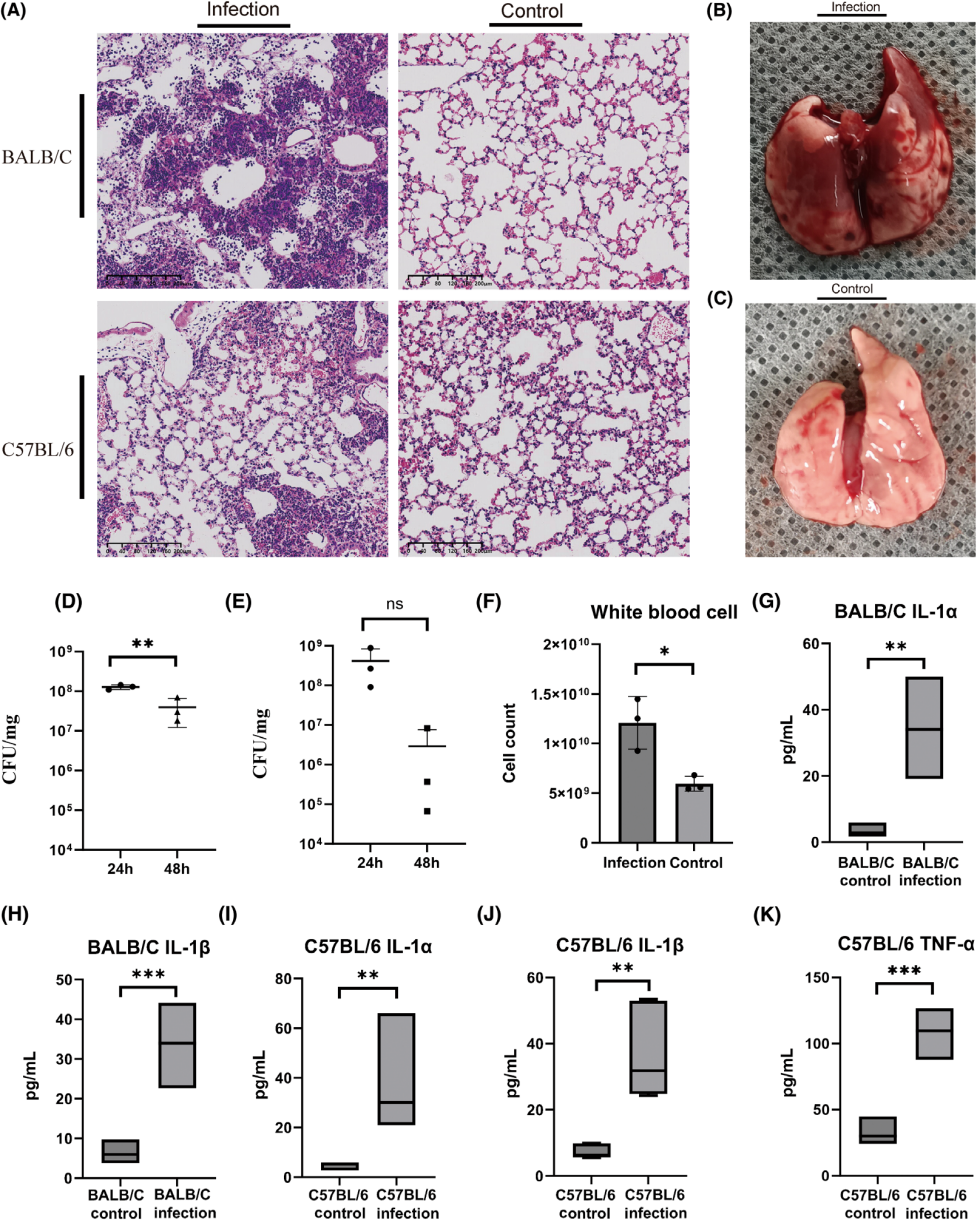
Infection model: (A) HE staining of control and infection groups. (B, C) Lungs of control and infection groups. (D) Lung bacterial load in BALB/c mice; (E) Lung bacterial load in C57BL/6 mice. (F) White blood cell count. (G, H) Inflammatory factors in BALB/c mice. (I–K) Inflammatory factors in C57BL/6 mice; *p < 0.05, **p < 0.01, ***p < 0.001, *n* = 3.

H&E staining revealed no discernible abnormalities in the lungs of the control group, while inflammatory cell aggregation was observed in the infection group (Figure 5A). Microscopic examination further confirmed that the lung tissues of the control group exhibited no significant pathological changes, whereas marked pulmonary injury was evident in the experimental group (Figure 5B,C). Bacterial loads in the lung tissues of C57BL/6 and BALB/c mice remained elevated 24 h post‐infection but showed a significant decrease at 48 h (Figure 5D,E). Hematological analysis showed that white blood cell counts were significantly increased in infected mice (Figure 5F). Levels of inflammatory factors IL‐1α and IL‐1β were significantly elevated in BALB/c mice (Figure 5G,H), while TNF‐α, IL‐1α, and IL‐1β were notably increased in the BALF of C57BL/6 mice (Figure 5I–K), confirming the successful establishment of the mouse infection model. A total of 28 samples were collected for dual PCR, qPCR, and LAMP‐LFD testing following the infection experiment. The multiplex PCR test detected 21 positive cases, whereas qPCR identified 23 positive cases (Table 4). Chi‐square analysis indicated no statistically significant differences among the three methods.

**TABLE 4:** Test results for the infection samples.

	Positive	Negative
Multiplex PCR	21	7
qPCR	23	5
LAMP‐LFD	19	9

### 3.6 Application in laboratory animal monitoring

**FIGURE 6:**
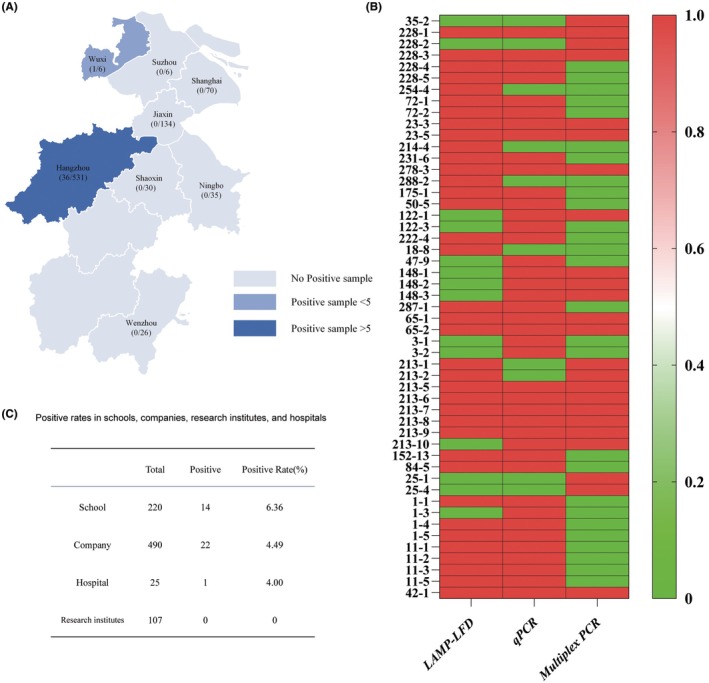
(A) Regional distribution of positive samples: the four samples from Guangdong are not shown. (B) Comparison of detection results for LAMP‐LFD, qPCR, and multiplex PCR across 51 clinical samples. Each row represents an individual sample, and each column corresponds to a detection method. Green indicates a positive result, and red indicates a negative result. (C) Positive rates across schools, companies, research institutes, and hospitals.

A total of 842 samples were analyzed. Positivity rates were 4.87% (41/842) by qPCR and 4.39% (37/842) by LAMP‐LFD (Table S1). Among the positive samples, 36 cases were detected in Hangzhou and 1 in Wuxi (Figure 6A). The diagnostic performance of the LAMP‐LFD assay was evaluated against qPCR and multiplex PCR. Visual inspection of the resulting heatmap is presented in Figure 6B. The LAMP–LFD assay showed excellent agreement with qPCR (*κ* = 0.785), moderate agreement with multiplex PCR (*κ* = 0.476), and low agreement with culture‐based detection (*κ* = 0.099) (Table 5). Additionally, positive cases were distributed as follows: 22 in companies, 14 in schools, and 1 in hospitals (Figure 6C).

**TABLE 5:** Results of the culture‐based, qPCR, multiplex PCR, and LAMP‐LFD assays.

	LAMP‐LFD			Coincidence rate (%)	Kappa value	*p*‐Value of kappa
	Positive	Negative	Total			
Culture‐based						
Positive	2	0	2			
Negative	35	805	840	95.84	0.099	0.51
Total	37	805	842			
qPCR						
Positive	31	10	41			
Negative	6	795	801	98.10	0.785	<0.001
Total	37	805	842			
Multiplex PCR						
Positive	15	9	24			
Negative	22	796	818	96.32	0.476	<0.001
Total	37	805	842			

## 4 DISCUSSION

Recent surveillance data from South Korea (2014–2019), encompassing 206 laboratory animal facilities, revealed a notable increase in infections caused by *R. heylii* and *R. pneumotropicus*. These species have emerged as among the most common bacterial contaminants, second only to *Staphylococcus aureus*. Both bacteria were detected in 12.5% of animals across conventional and barrier housing systems. A similar trend was observed in Japan, where the overall contamination rate in laboratory mice during the same period averaged 4.6%.^28^ Testing conducted by Suzhou Xishan Biotechnology in China in 2021 reported an infection rate of 4.5%.

Multiple investigations have documented the isolation of *R. heylii* and *R. pneumotropicus* from environmental sources and laboratory animals.^29^, ^30^, ^31^ Collectively, these findings indicate that both species are widely distributed in laboratory animal environments and pose a considerable risk, particularly to immunodeficient or SPF animals.

This study introduces and validates a LAMP‐LFD approach for the specific identification of *R. heylii* and *R. pneumotropicus*. By targeting the 16S‐23S rRNA ITS region, which exhibits sufficient interspecies variability, the assay allows for species‐specific primer design.^24^ Validation results demonstrated high sensitivity and specificity, showing 98.10% concordance with qPCR across 842 clinical samples. The assay's sensitivity was further confirmed using simulated clinical samples spiked with serial dilutions of the target bacteria. The LoD were shown to be as low as 10^2^ CFU/mL for *R. heylii* and 10^1^ CFU/mL for *R. pneumotropicus* in throat swab samples, and 10^1^ and 10^0^ CFU/mL in fecal samples, respectively. The slightly lower detection rate of LAMP‐LFD compared to qPCR may be attributed to low pathogen abundance in some clinical samples; however, spiked‐sample experiments confirm that the assay remains reliable even at very low bacterial loads. Moreover, the closed‐tube design effectively minimizes the risk of aerosol contamination, simplifies result interpretation, and eliminates the need for specialized equipment or technical expertise.

The clinical relevance of these pathogens was further validated using an animal infection model. Infected mice exhibited characteristic symptoms, including weight loss and respiratory distress, alongside successful pulmonary colonization. The results showed that infection triggered a pulmonary inflammatory response, as evidenced by a significant increase in IL‐1β and TNF‐α levels in BALF.^4^, ^32^, ^33^ These findings not only underscore the pathogenic potential of these bacteria but also emphasize the importance of accurate monitoring in laboratory animal populations.

Several limitations warrant consideration. First, while the method demonstrated strong specificity against closely related species, encompassing *R. ratti*, *Pasteurella*, and other members of the *Pasteurellaceae* family, the lack of reference strains for all known *Rodentibacter* species (e.g., *R. haemolyticus, R. abscessus*)^32^, ^33^ precludes a comprehensive assessment of cross‐reactivity. Second, the observed cross‐reactivity between primer sets for *R. heylii* and *R. pneumotropicus* reflects both the genetic homology between these species and the limitations of ITS‐based discrimination. However, the concurrent use of both primer sets provided complete coverage of all target strains, providing a practical diagnostic solution.

Culture‐based detection showed markedly lower positivity than molecular assays, with only two culture‐positive samples identified among 842 specimens. This discrepancy likely reflects low bacterial abundance, uneven respiratory distribution, and the fastidious growth characteristics of *Rodentibacter* spp., which may lead to underestimation by conventional culture.^34^ In contrast, nucleic acid‐based methods detect bacterial DNA independently of viability, explaining the stronger agreement observed between LAMP–LFD and qPCR.^35^, ^36^ The comparatively lower agreement with multiplex PCR may be attributable to primer competition and subjective interpretation of closely sized electrophoretic bands.

Because LAMP produces large quantities of amplification products, strict contamination‐control practices are essential. Physical separation of workflow areas, avoidance of post‐amplification tube opening, and inclusion of appropriate negative controls were implemented in this study, and the closed‐tube detection design effectively reduced carryover contamination and improved assay reliability.

Despite these limitations, the LAMP‐LFD method remains a robust screening tool for primary laboratories, notable for its minimal equipment requirements and operational simplicity. Epidemiological analysis of 842 clinical samples revealed elevated pathogen prevalence in educational institutions, highlighting the need for enhanced biosafety protocols, including standardized rearing practices, systematic serological screening, and preventive quarantine measures to mitigate *Rodentibacter* transmission.

Future efforts should aim to expand specificity validation by including additional *Rodentibacter* strains and integrating rapid DNA extraction protocols to enable true sample‐to‐result testing. Rapid bacterial DNA extraction techniques have been described previously^37^ and offer advantages such as reduced processing time and simplified workflows. Building on this concept, future work will focus on developing protocols capable of isolating DNA directly from tracheal tissues for application with the LAMP‐LFD assay. These improvements would enhance the practicality of the method in resource‐limited settings and further strengthen both laboratory animal health surveillance and the reliability of experimental outcomes.

## 5 CONCLUSION

This study aimed to develop an on‐site rapid detection technique, leading to the successful establishment of the LAMP‐LFD detection method. Cross‐reactivity assessment showed that the system did not react with 14 common symbiotic or pathogenic bacteria and exhibited a detection limit of 1 × 10^−5^ ng/μL. Its analytical sensitivity exceeded that of conventional PCR, allowing reliable identification of *R. heylii* and *R. pneumotropicus*. Following sample loading, visual readouts were obtained within approximately 15 min, and the complete workflow required no more than 90 min. Owing to its simplicity, rapid turnaround, and minimal equipment requirements, this approach offers a practical solution for routine screening in basic laboratory settings.

## AUTHOR CONTRIBUTIONS

**Huiqiong Yan:** Investigation; methodology; writing – original draft. **Sisi Chen:** Investigation; methodology; writing – original draft. **Yuhan Gan:** Data curation. **Jing Xing:** Methodology. **Yufang Feng:** Data curation; formal analysis. **Xin Wen:** Data curation; formal analysis. **Jie Fang:** Methodology. **Jiangtao Du:** Formal analysis. **Shasang Zhou:** Data curation. **Honggang Guo:** Data curation; formal analysis. **Xiaoyin Jin:** Methodology. **Zhiyuan Wang:** Methodology. **Junhao Tao:** Methodology. **Lingqun Lu:** Methodology. **Qingming Kong:** Data curation. **Huazhong Ying:** Supervision. **Wei Han:** Conceptualization; supervision. **Fangwei Dai:** Conceptualization; project administration; writing – review and editing.

## FUNDING INFORMATION

This study was funded by the Medical Science and Technology Project of Zhejiang Province (grant no. 2018KY351 and 2020KY530), Scientific research project of Zhejiang Graduate Education Society (grant no. 2021009 and 2023027), Basic Public Welfare Research Program of Zhejiang Province (grant no. LGD22C040024 and ZCLTGD24C0401), Zhejiang Traditional Chinese Medicine Administration (grant no. 2021ZB081), Department of Education of Zhejiang Province (grant no. Y201942573), Project of the Central Government leading Local Scientific and Technological Development Fund (2025ZY01054), Project of Zhejiang Key Laboratory (2025E10040). Postgraduate Research Project and construction project of Hangzhou Medical College (26100548300‐6: 26100548300‐10).

## CONFLICT OF INTEREST STATEMENT

The authors declare that they have no conflict of interest.

## ETHICS STATEMENT

All work using animals was approved by the Ethics Committee for Experimental Animal Welfare of Zhejiang Center of Laboratory Animal (reference No. ZJCLA‐IACUC‐20020119), all applicable guidelines for the care and use of animals were followed.

## Supporting information


**Figure S1.** Optimization of deoxynucleotide triphosphate (dNTP) and magnesium (Mg^2+^) concentrations in the loop‐mediated isothermal amplification (LAMP) reaction systems for *Rodentibacter heylii* and *Rodentibacter pneumotropicus*. (A, B) Optimization of dNTP and Mg^2+^ concentrations in the *R. pneumotropicus* LAMP reaction system. (C, D) Optimization of dNTP and Mg^2+^ concentrations in the *R. heylii* LAMP reaction system.
**Figure S2.** Temperature optimization of the *Rodentibacter heylii* and *Rodentibacter pneumotropicus* loop‐mediated isothermal amplification reaction system: temperatures tested were 61°C, 62°C, 63°C, 64°C, 65°C, and 66°C. Three sample types (*R. pneumotropicus*, *R. heylii*, and a negative control) were tested, and each condition was performed in triplicate.
**Table S1.** Sources of Clinical Samples and positivity rates of the LAMP‐LFD assay.

## Data Availability

These datasets analyze during the current study are available in the Genbank repository (Genbank JX010710.1 and JX010706.1).
