# Supplementary material for: Development of a loop‐mediated isothermal amplification‐lateral flow dipstick (LAMP‐LFD) assay and on‐site rapid detection evaluation for Rodentibacter heylii and Rodentibacter pneumotropicus
Source: Animal Model Exp Med. 2026 May 28;9(7):1395–408. doi: 10.1002/ame2.70214 (PMC13394658; doi:10.1002/ame2.70214)
Supplement: Supplementary file 1 — Figure S1. Optimization of deoxynucleotide triphosphate (dNTP) and magnesium (Mg2+) concentrations in the loop‐mediated isothermal amplification (LAMP) reaction systems for Rodentibacter heylii and Rodentibacter pneumotropicus. (A, B) Optimization of dNTP and Mg2+ concentrations in the R. pneumotropicus LAMP reaction system. (C, D) Optimization of dNTP and Mg2+ concentrations in the R. heylii LAMP reaction system. Figure S2. Temperature optimization of the Rodentibacter heylii and Rodentibacter pneumotropicus loop‐mediated isothermal amplification reaction system: temperatures tested were 61°C, 62°C, 63°C, 64°C, 65°C, and 66°C. Three sample types (R. pneumotropicus, R. heylii, and a negative control) were tested, and each condition was performed in triplicate. Table S1. Sources of Clinical Samples and positivity rates of the LAMP‐LFD assay. [file AME2-9-1395-s001.docx]

**
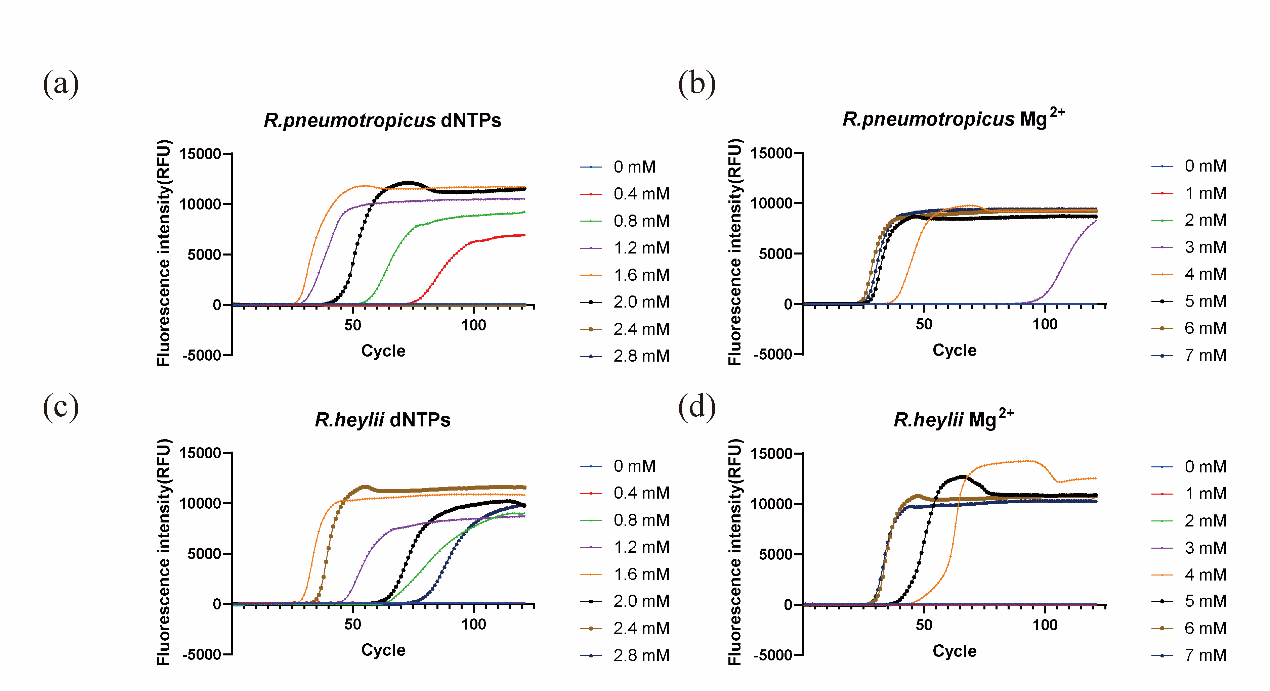
Fig S1** Optimization of deoxynucleotide triphosphate (dNTP) and magnesium (Mg²⁺) concentrations in the loop-mediated isothermal amplification (LAMP) reaction systems for *Rodentibacter heylii* and *Rodentibacter pneumotropicus*. (a–b) Optimization of dNTP and Mg²⁺ concentrations in the *R. pneumotropicus* LAMP reaction system. (c–d) Optimization of dNTP and Mg²⁺ concentrations in the *R. heylii* LAMP reaction system.


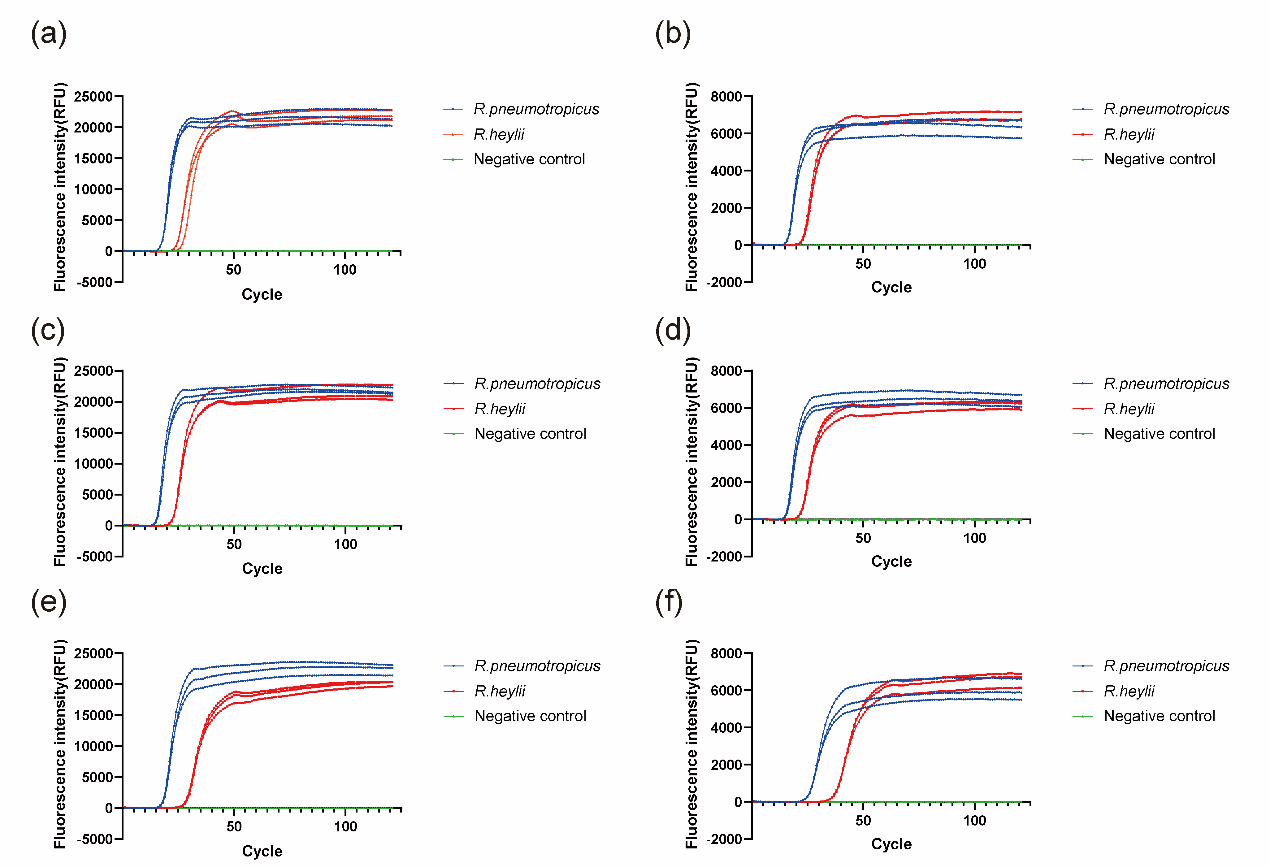


**Fig S2** Temperature optimization of the *Rodentibacter heylii* and *Rodentibacter pneumotropicus* loop-mediated isothermal amplification reaction system: temperatures tested were 61°C, 62°C, 63°C, 64°C, 65°C, and 66°C. Three sample types (*R. pneumotropicus*, *R. heylii*, and a negative control) were tested, and each condition was performed in triplicate.

**Table S1.** Sources of Clinical Samples and positivity rates of the LAMP-LFD assay.

| Number of positive samples/number of total samples (%) | | | |
| --- | --- | --- | --- |
| Animal | LAMP-LFD | qPCR | Multiplex PCR |
| ICR mice | 5/157 | 5/157 | 2/157 |
| C57BL/6 mice | 7/178 | 7/178 | 3/178 |
| Sentinel mice | 0/46 | 0/46 | 0/46 |
| SCID beige mice | 0/1 | 0/1 | 0/1 |
| Rats (Not classified)） | 1/5 | 0/5 | 0/5 |
| BALB/c mice | 2/61 | 2/61 | 2/61 |
| F1 mice | 0/16 | 0/16 | 0/16 |
| CD-1 mice | 0/20 | 0/20 | 0/20 |
| CD Rats | 0/24 | 0/24 | 0/24 |
| Wistar Rats | 0/40 | 0/40 | 0/40 |
| SD Rats | 1/54 | 1/54 | 0/54 |
| nude mice | 0/48 | 0/48 | 0/48 |
| Apoe mice | 0/7 | 0/7 | 0/7 |
| KM mice | 0/16 | 0/16 | 0/16 |
| DB.DB mice | 0/1 | 0/1 | 0/1 |
| Golden Ground Squirrels | 1/7 | 7/7 | 4/7 |
| Cavy | 1/10 | 1/10 | 1/10 |
| Tg(Syn1-cre)671Jxm mice | 0/1 | 0/1 | 0/1 |
| Tg(GFAP-cre)8Gtm mice | 0/1 | 0/1 | 0/1 |
| REST mice | 0/1 | 0/1 | 0/1 |
| Mice (Not classified)） | 12/12 | 10/126 | 11/126 |
| NOD.SCID mice | 0/1 | 0/1 | 0/1 |
| Eriones unguiculatus | 0/5 | 0/5 | 0/5 |
| Transgenic mice | 7/15 | 8/15 | 1/15 |
| APOJ mice | 0/1 | 0/1 | 0/1 |
| Overall  (n=842) | 37/842  (4.39) | 41/842  (4.87) | 24/842  (2.73) |
